# SIRT1 Promotes Host Protective Immunity against *Toxoplasma gondii* by Controlling the FoxO-Autophagy Axis via the AMPK and PI3K/AKT Signalling Pathways

**DOI:** 10.3390/ijms232113578

**Published:** 2022-11-05

**Authors:** Jina Lee, Jinju Kim, Jae-Hyung Lee, Yong Min Choi, Hyeonil Choi, Hwan-Doo Cho, Guang-Ho Cha, Young-Ha Lee, Eun-Kyeong Jo, Byung-Hyun Park, Jae-Min Yuk

**Affiliations:** 1 Department of Infection Biology, College of Medicine, Chungnam National University, Daejeon 35015, Korea; 2 Infection Control Convergence Research Center, College of Medicine, Chungnam National University, Daejeon 35015, Korea; 3 Department of Medical Science, College of Medicine, Chungnam National University, Daejeon 35015, Korea; 4 Department of Microbiology, College of Medicine, Chungnam National University, Daejeon 35015, Korea; 5 Department of Biochemistry, Chonbuk National University Medical School, Jeonju 54896, Korea

**Keywords:** Sirtuin 1, autophagy, AMP-activated protein kinase, PI3K/AKT signalling pathway, *Toxoplasma gondii*, bone-marrow-derived macrophages, Class O of forkhead box transcription factors

## Abstract

Sirtuin 1 (SIRT1) regulates cellular processes by deacetylating non-histone targets, including transcription factors and intracellular signalling mediators; thus, its abnormal activation is closely linked to the pathophysiology of several diseases. However, its function in *Toxoplasma gondii* infection is unclear. We found that SIRT1 contributes to autophagy activation via the AMP-activated protein kinase (AMPK) and PI3K/AKT signalling pathways, promoting anti-*Toxoplasma* responses. Myeloid-specific *Sirt1*^−/−^ mice exhibited an increased cyst burden in brain tissue compared to wild-type mice following infection with the avirulent ME49 strain. Consistently, the intracellular survival of *T. gondii* was markedly increased in *Sirt1*-deficient bone-marrow-derived macrophages (BMDMs). In contrast, the activation of SIRT1 by resveratrol resulted in not only the induction of autophagy but also a significantly increased anti-*Toxoplasma* effect. Notably, SIRT1 regulates the FoxO-autophagy axis in several human diseases. Importantly, the *T. gondii*-induced phosphorylation, acetylation, and cytosolic translocation of FoxO1 was enhanced in *Sirt1*-deficient BMDMs and the pharmacological inhibition of PI3K/AKT signalling reduced the cytosolic translocation of FoxO1 in BMDMs infected with *T. gondii*. Further, the CaMKK2-dependent AMPK signalling pathway is responsible for the effect of SIRT1 on the FoxO3a-autophagy axis and for its anti-*Toxoplasma* activity. Collectively, our findings reveal a previously unappreciated role for SIRT1 in *Toxoplasma* infection.

## 1. Introduction

*Toxoplasma gondii* is an opportunistic obligate intracellular protozoan parasite that is capable of infecting all nucleated cells of a variety of warm-blooded animals; it infects approximately one-third of the world’s population [1,2]. *Toxoplasma* infection of immunocompetent individuals is typically asymptomatic; however, >10% of infected individuals develop cervical lymphadenopathy and toxoplasmic chorioretinitis [3]. In addition, toxoplasmosis in immunocompromised patients, including those on immunosuppressive drugs or chemotherapy, those with AIDS [4], and congenitally infected individuals, can be life-threatening because of toxoplasmic encephalitis or pneumonitis [5,6]. During *Toxoplasma* infection, parasite proliferation in innate immune cells is promoted by the remodelling of the phagosomal compartments via parasitophorous vacuole (PV) membrane formation and the subsequent production of diverse antigens [7]. Moreover, *T. gondii* interferes with the activation of host immunity by suppressing the inflammatory response and the production of microbicidal nitric oxide and cytokines [8,9].

Autophagy is an essential biological process for the degradation of cytosolic components, including organelles and soluble macromolecules, by lysosomal digestion [10]. Autophagy-related genes (Atgs) and autophagy-regulating kinases are important components of the autophagy process. The UNC-51-like kinase 1/2 (ULK1/2) complex is a key protein in the initiation of autophagy and is regulated by AMP-activated protein kinase (AMPK) and mammalian target of rapamycin complex 1 (mTORC1) [10,11]. Together with maintaining cellular homeostasis, autophagy is implicated in activating an immune response to *Toxoplasma* infection [12,13,14]. *T. gondii* has various strategies to preserve PV integrity by attenuating host–cell signalling related to the autophagic targeting of PV and activating mTORC1 via the PI3K/AKT signalling axis [12]. Therefore, additional studies on their interactions are needed.

Sirtuin 1 (SIRT1) is a nicotinamide adenosine dinucleotide (NAD)-dependent protein deacetylase that participates in a wide range of biological processes, including metabolism, cell cycle, inflammation, neurodegeneration, and DNA repair [15,16]. SIRT1 deacetylates various histone and non-histone proteins, such as the transcription factor forkhead box class O (FoxO), the tumor suppressor p53, and nuclear factor κB (NF-κB), to regulate cellular physiology and energy expenditure [17]. SIRT1 directly and indirectly modulates autophagy. The SIRT1-mediated deacetylation of FOXO1 induces autophagy by inducing the expression of small GTPase Rab 7 [18], which is required for the fusion of autophagosomes and terminal endosomes/lysosomes [19]. Moreover, the SIRT1-dependant deacetylation of FOXO3 induces BCL2/adenovirus E1B 19 kDa protein-interacting protein 3 (Bnip3)-mediated autophagy [20]. SIRT1 also deacetylates Atg5, 7, and 8 [21]. The deacetylation of microtubule-associated protein 1 light-chain 3 (LC3) by SIRT1 results in relocation from the nucleus to autophagosomes in the cytoplasm under starvation conditions, followed by autophagy activation [22]. In addition, SIRT1 activation by resveratrol (RES) activated autophagy and attenuated apoptosis by modulating AMPK-dependent nutrient-sensing pathways, thus improving spinal cord injury [23]. Previously, we reported that 4-hydroxybenzaldehyde (4-HBA) suppresses the growth of intracellular *T. gondii* by SIRT1-dependent the activation of autophagy [24]. Although SIRT1 regulates diverse cellular processes, its role in the modulation of host protective immunity during *Toxoplasma* infection is unclear.

In this study, myeloid-specific *Sirt1*^−/−^ mice (m*Sirt1*^−/−^ mice) had a markedly higher protozoan parasite load and *T. gondii* surface antigen 1 (*Sag1*) mRNA level in brain tissues. Moreover, a *Sirt1* deficiency in primary macrophages increased the survival and proliferation of intracellular *T. gondii*. Conversely, the pharmacological activation of SIRT1 induced autophagy activation and then promoted the host protective immune response against *Toxoplasma* infection. In addition, RES-mediated autophagy induction, autophagosome maturation, and lysosomal fusion were diminished in *Sirt1*-deficient primary macrophages. Finally, the activities of FoxO1 and FoxO3a, key regulators of autophagy, were modulated by SIRT1 via the PI3K/AKT and CAMKK2/AMPK signalling pathways, respectively. These data indicate a previously unappreciated function for myeloid SIRT1 in the regulation of autophagy and anti-*Toxoplasma* responses in mouse primary macrophages.

## 2. Results

### 2.1. Myeloid SIRT1 Is Required for Antiparasitic Activity in Response to Toxoplasma Infection

**Figure 1:**
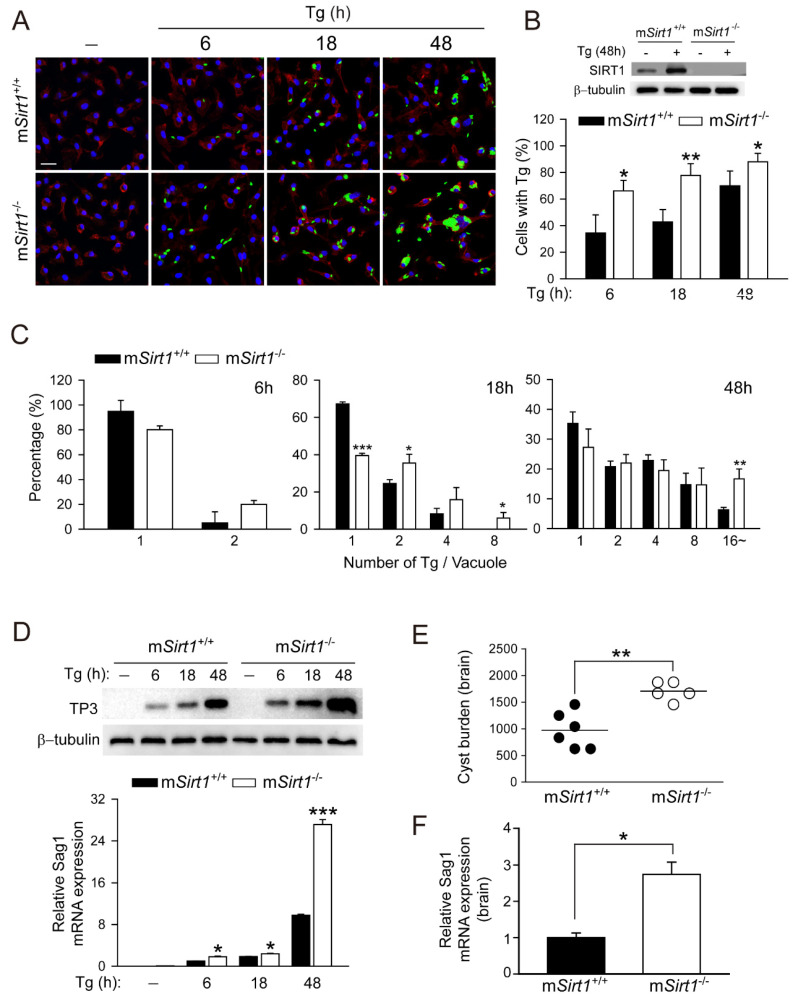
Myeloid SIRT1 is essential for host protection against *T. gondii* infection in primary macrophages and mice. (**A**–**C**) BMDMs from m*Sirt1^+/+^* and m*Sirt1*^−/−^ mice were infected with GFP-conjugated *T. gondii* RH strain (MOI = 1) for the indicated periods. Cells were fixed and stained with Texas Red^®^-X phalloidin for F-actin in the cytoskeleton (red) and DAPI for nuclei (blue), respectively. (**A**) Fluorescent images showing the number of intracellular *T. gondii*. (**B**,**C**) Number of *T. gondii* RH-infected cells (for **B**) and *T. gondii* RH per vacuole (for **C**) were quantified. Scale bar = 25 µm (**D**) BMDMs from m*Sirt1^+/+^* and m*Sirt1*^−/−^ mice were infected with *T. gondii* RH strain (MOI = 1) for the indicated time periods. TP3 protein expression (top) and *Sag1* mRNA expression (bottom) was examined by immunoblot and qPCR analysis, respectively. (**E**,**F**) m*Sirt1^+/+^* and m*Sirt1*^−/−^ mice (n = 5 per group) were infected with 40 cysts of the ME49 strain (i.p. injection) for 3 weeks. (**E**) The number of cysts in the brain was counted under a microscope. (**F**) *Sag1* mRNA expression in brain homogenates was evaluated by real-time qPCR analysis. Data are representative of three independent experiments and are presented as means ± SD (for **B**–**D**) or ± SEM (for **F**). * *p* < 0.05, ** *p* < 0.01, *** *p* < 0.001, compared with control cultures or control mice infected with *T. gondii* (two-tailed Student’s *t*-test). Tg, *Toxoplasma gondii*.

We reported that 4-HBA exerts an anti-*Toxoplasma* effect by inducing autophagy in a manner dependent on SIRT1 [24]. Herein, we examined whether SIRT1 contributes to the activation of host defences against *T*. *gondii* infection. To assess the role of myeloid-specific SIRT1, BMDMs were isolated from m*Sirt1*^+/+^ or m*Sirt1*^−/−^ mice and infected with a GFP-expressing RH strain of *T. gondii* (GFP-Tg) for the indicated time periods (Figure 1A–C). The number of cells infected with *T. gondii* (Figure 1A,B) and the proliferation of intracellular *T. gondii* (Figure 1A,C) were significantly enhanced in *Sirt1*-deficient BMDMs compared to wild-type (WT) BMDMs. The levels of the *Toxoplasma* antigens p30 membrane protein (for TP3 protein; Figure 1D, top) and *Sag1* mRNA (Figure 1D, bottom) were markedly increased in *Sirt1*-deficient BMDMs.

We next examined the in vivo role of myeloid-specific SIRT1 in response to *Toxoplasma* infection. m*Sirt1^+/+^* and m*Sirt1*^−/−^ mice were intraperitoneally infected with strain type II ME49 (Figure 1E; 40 cyst/mouse), respectively. The parasite load in infected brain tissue was significantly greater in m*Sirt1*^−/−^ mice than in *mSirt1^+/+^* mice (Figure 1E). In addition, the mRNA level of *Sag1* was increased in the infected brains of m*Sirt1*^−/−^ mice (Figure 1F). Taken together, these data suggest that myeloid-specific SIRT1 plays a pivotal role in the antiparasitic response to *Toxoplasma*.

### 2.2. RES Restricts Intracellular Parasite Growth by Activating SIRT1 in Primary Macrophages

**Figure 2:**
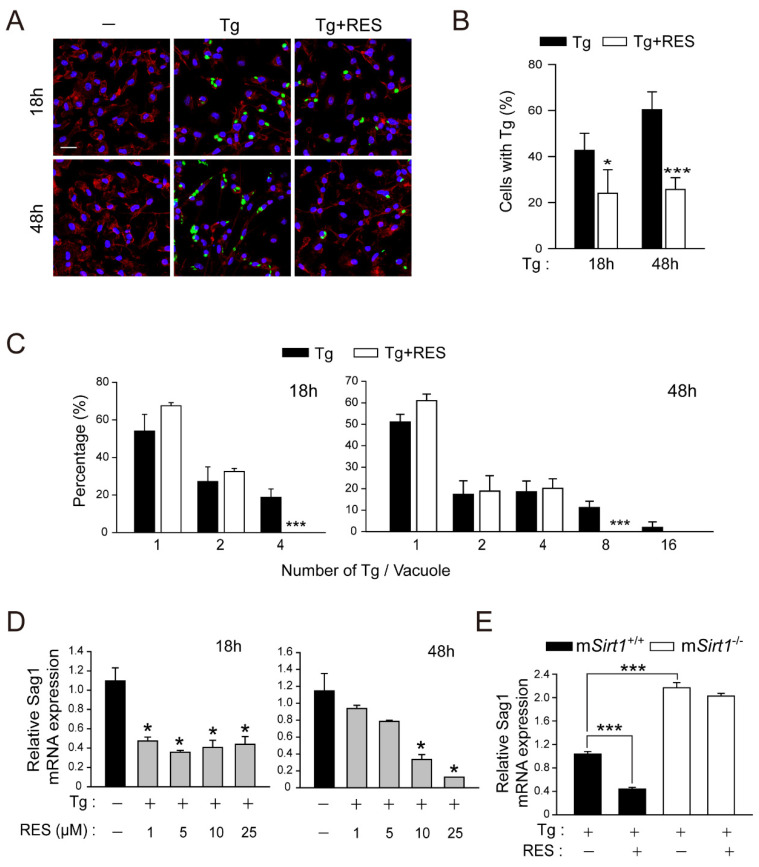
RES treatment promotes the elimination of intracellular *T. gondii* in primary murine macrophages. (**A**–**C**) BMDMs were infected with a GFP-conjugated RH strain (MOI = 1) for 2 h, followed by the further treatment of RES (10 µM) for the indicated times. Cells were stained with Texas Red^®^-X phalloidin for F-actin in the cytoskeleton (red) and DAPI for nuclei (blue), respectively. (**A**) Fluorescent images showing the number of intracellular *T. gondii*. (**B**,**C**) Number of *T. gondii* RH-infected cells (for **B**) and *T. gondii* RH per vacuole were quantified. Scale bar = 25 µm (**D**,**E**) qPCR analysis for evaluating *Sag1* mRNA expression. (**D**) *T. gondii*-infected BMDMs were stimulated with increasing concentrations of RES (1, 5, 10, or 25 µM) for 18 h (for left) or 48 h (for right). (**E**) BMDMs isolated from m*Sirt1^+/+^* and m*Sirt1*^−/−^ mice were infected with RH strain of *T. gondii* (MOI = 1) for 2 h and then further stimulated with RES (10 µM) for 48 h. Data are representative of three independent experiments and are presented as means ± SD. * *p* < 0.05 and *** *p* < 0.001, compared with control cells (two-tailed Student’s *t*-test). Tg, *Toxoplasma gondii*; RES, resveratrol.

The stilbenoid polyphenol RES (trans 3,5,4′-trihydroxystilbene) exerts beneficial effects by regulating SIRT1 activity in a range of cellular functions [25]. To determine whether the activation of SIRT1 by RES induces antiparasitic responses in macrophages, we compared the number of cells infected with *T. gondii* (Figure 2A,B) and the intracellular proliferation of *T. gondii* (Figure 2A,C). Contrary to the effect of a myeloid-specific SIRT1 deficiency (Figure 1), the intracellular growth of *T. gondii* was markedly reduced in cells treated with RES compared to the solvent control. Similarly, RES significantly decreased the *Sag1* mRNA level in BMDMs infected with *T. gondii* (Figure 2D). To investigate the role of SIRT1 in the anti-*Toxoplasma* effect of RES, we compared the mRNA level of *Sag1* after RES treatment in m*Sirt1^+/+^* and m*Sirt1*^−/−^ BMDMs infected with *T. gondii*. RES inhibited *T. gondii* proliferation in m*Sirt1^+/+^* BMDMs (Figure 2E). However, RES did not exert an anti-*Toxoplasma* effect in *Sirt1*-deficient primary macrophages. Collectively, these data suggest that SIRT1 is required for the effect of RES on anti-*Toxoplasma* activity.

### 2.3. Pharmacological Activation of SIRT1 by RES Is Essential for the Induction of Antiprotozoal Autophagy and the Colocalisation of PV with Autophagosomes/Lysosomes

**Figure 3:**
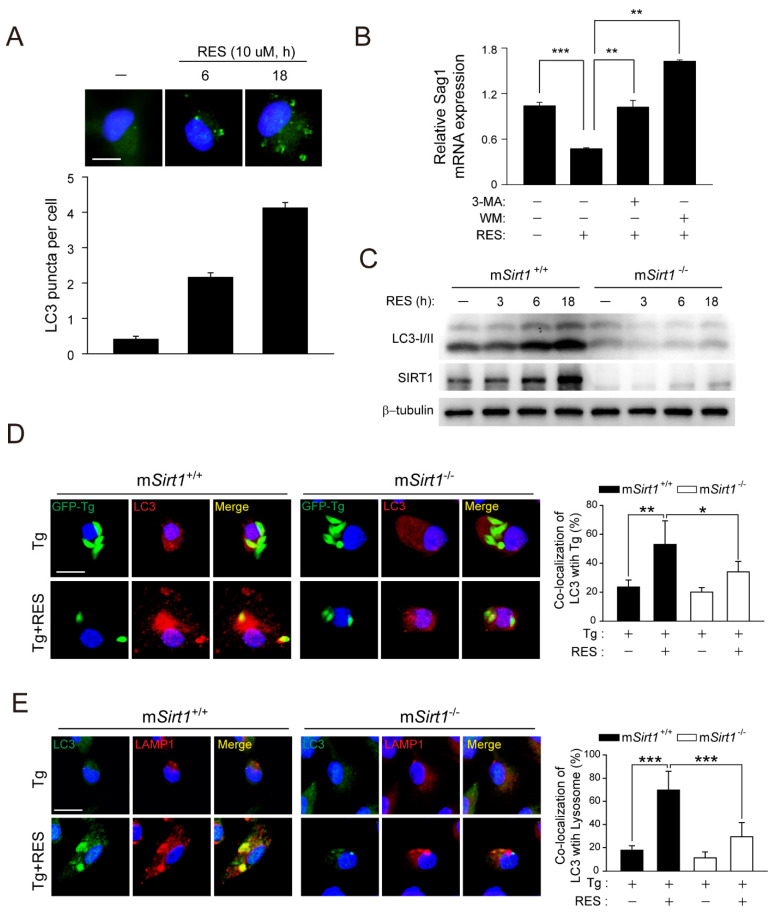
RES induces autophagy activation in a SIRT1-dependent manner. (**A**) BMDMs were stimulated with RES (10 µM) for various times and subjected to immunofluorescence microscopy for analysing LC3 puncta formation (top). Quantification of LC3 punctate foci per cell (bottom). Each experiment included a minimum of 100 cells scored in 5 random fields. Scale bar = 10 µm. (**B**) BMDMs were pre-treated with 3-MA (10 µM, 2 h) or WM (100 nM, 2 h) and further incubated with RES for 18 h. The mRNA expression of the *Sag1* gene was evaluated by real-time qPCR analysis. (**C**) BMDMs from m*Sirt1^+/+^* and m*Sirt1*^−/−^ mice were stimulated with RES for the indicated time periods and subjected to immunoblot analysis of LC3 and β-tubulin. (**D**,**E**) BMDMs from m*Sirt1^+/+^* and m*Sirt1*^−/−^ mice were infected with a GFP-conjugated RH strain (for **D**) or a *T. gondii* RH strain (for **E**) for 2 h, followed by further treatment of RES (10 µM) for 18 h. (**D**) Cells were immunostained with an anti-LC3 antibody (Alexa Fluor 594-conjugated goat anti-rabbit IgG, red) and the level of colocalisation *T. gondii* with LC3 was quantified. Scale bar = 10 µm. (**E**) LC3 (Alexa Fluor 488-conjugated goat anti-rabbit IgG, green), Alexa Fluor 594-conjugated LAMP1 (red), and DAPI (blue) were detected by confocal microscopy. Immunofluorescence microscopy images were obtained from one representative of 3 independent samples, with each experiment containing a minimum of 50 cells scored in 7 random fields. Scale bar = 10 µm. Data are representative of three independent experiments and are presented as means ± SD. * *p* < 0.05, ** *p* < 0.01, and *** *p* < 0.001, compared with control cells (two-tailed Student’s *t*-test). Tg, *Toxoplasma gondii*; RES, resveratrol; 3-MA, 3-methyladenine; WM, wortmannin.

SIRT1 modulates health and disease by modulating autophagy activation via the transcriptional or epigenetic regulation of Atgs and autophagy regulators [26]. To determine whether RES induces autophagy, we enumerated LC3 punctate structures in BMDMs. RES significantly increased the number of LC3 aggregates in a time-dependent manner (Figure 3A). Moreover, the RES-mediated decrease in *Sag1* mRNA levels was significantly attenuated by pretreatment with the autophagy inhibitors 3-methyladenine (3-MA) and wortmannin (WM) (Figure 3B). However, the RES-mediated increase in the levels of LC3-II proteins was suppressed in m*Sirt1*-deficient BMDMs (Figure 3C).

Based on the above, we next questioned whether the pharmacological activation of SIRT1 by RES treatment is involved in autophagosome maturation and lysosomal degradation in BMDMs infected with *T. gondii*. RES significantly enhanced the intracellular colocalisation of LC3-positive autophagic vacuoles with *T. gondii*-containing PVs (Figure 3D) and autophagolysosomal fusion (Figure 3E), which was significantly decreased in m*Sirt1*-deficient BMDMs. Therefore, SIRT1 mediated RES-induced autophagy activation and antiparasitic responses in primary macrophages.

### 2.4. SIRT1 Suppresses T. gondii-Induced FoxO1 Activation Via the PI3K/AKT Signalling Pathway in T. gondii Infection

**Figure 4:**
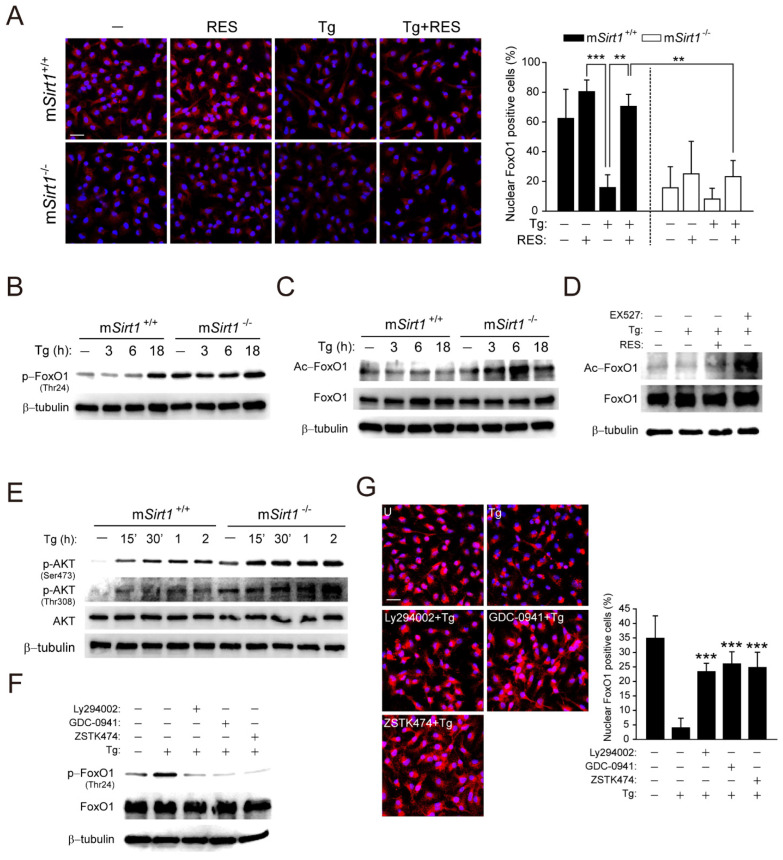
SIRT1 deficiency leads to the excessive activation of PI3K/AKT-mediated FoxO1 upon *T. gondii* infection. (**A**) BMDMs from m*Sirt1^+/+^* and m*Sirt1*^−/−^ mice were infected with a RH strain (MOI = 1, for 2 h) and then further stimulated with RES (10 µM) for 18 h. Cells were immunostained using an anti-FoxO1 antibody (Alexa Fluor 568-conjugated goat anti-rabbit IgG, red) and DAPI for nuclei (blue). Cells were then subjected to immunofluorescence microscopy. Representative images (left) and quantitative data (right) showing the cytoplasmic translocation of FoxO1 are from one representative of 3 independent samples, with each experiment containing a minimum of 50 cells scored in 3 random fields. Scale bar = 25 µm. (**B**,**C**,**E**) BMDMs from m*Sirt1^+/+^* and m*Sirt1*^−/−^ mice were infected with *T. gondii* for the indicated time periods. The phosphorylation (for **B**) and acetylation (for **C**) of FoxO1 or AKT phosphorylation (Ser473 and Thr308, for **E**) were determined by immunoblot analysis. (**D**) BMDMs were infected with *T. gondii* for 18 h in the presence of RES (10 µM) or EX527 (4 µM). The level of FoxO1 acetylation was evaluated by immunoblot analysis. (**F**,**G**) BMDMs were infected with *T. gondii* for 18 h in the presence of LY294002 (10 µM), GDC0941 (250 nM), or ZSTK474 (10 nM). (**F**) The level of FoxO1 phosphorylation was evaluated by immunoblot analysis, and total protein was determined by monitoring β-tubulin (**B**–**F**), AKT (**E**), or FoxO1 (**C**,**D**,**F**) as a loading control. (**G**) Immunofluorescence microscopy analysis was assessed to determine the nuclear and cytoplasmic localisation of FOXO1, as described in Figure 4A. Scale bar = 25 µm. Data are representative of three independent experiments and are presented as means ± SD. ** *p* < 0.01 and *** *p* < 0.001, compared with m*Sirt1^+/+^* cells (for **A**) or control cells (for **G**) (two-tailed Student’s *t*-test). Tg, *Toxoplasma gondii*; RES, resveratrol.

SIRT1 is involved in FoxO1-mediated autophagy regulation, which has been implicated in tissue homeostasis, cellular stress, carcinogenesis, and immunity [18,26,27]. To examine the effect of SIRT1 on FoxO1 activity during *T. gondii* infection, we evaluated cytosolic translocation, phosphorylation, and acetylation in m*Sirt1^+/+^* and m*Sirt1*^−/−^ BMDMs (Figure 4A–C). *Toxoplasma gondii* infection promoted FoxO1 cytoplasmic translocation in m*Sirt1^+/+^* BMDMs, but this was abolished by post-treatment with RES (Figure 4A). Moreover, the proportion of nuclear FoxO1-positive cells in normal m*Sirt1*^−/−^ BMDMs was similar to that in m*Sirt1^+/+^* BMDMs infected with *T. gondii*. The inhibition by RES of *T. gondii*-induced FoxO1 cytosolic translocation was not observed in m*Sirt1*^−/−^ BMDMs. Furthermore, *T. gondii*-induced phosphorylation (Figure 4B) and acetylation (Figure 4C) of FoxO1 were enhanced in m*Sirt1*^−/−^ compared to m*Sirt1^+/+^* BMDMs. Similarly, stimulation with EX527, a selective SIRT1 inhibitor, increased FoxO1 acetylation in BMDMs infected with *T. gondii*, but stimulation with RES had no effect (Figure 4D).

AKT-induced phosphorylation of FoxO1 promotes its exclusion from the nucleus, leading to attenuated transcription of genes associated with autophagy [27,28]. *Toxoplasma gondii* rapidly induced the phosphorylation of AKT (Ser473 and Thr308) within 15 min in m*Sirt1^+/+^* BMDMs, and the expression level was greater in m*Sirt1*^−/−^ BMDMs (Figure 4E). *Toxoplasma gondii*-induced FoxO1 phosphorylation (Figure 4F) and cytosolic translocation (Figure 4G) and AKT phosphorylation (Figure S1) were attenuated by selective inhibitors of PI3K/AKT signalling, such as Ly294002, GDC-0941, or ZSTK474. These results indicate that a deficiency of myeloid SIRT1 promotes the *T. gondii*-induced hyperactivation of PI3K/AKT signalling, leading to impaired FoxO1 activation in primary murine macrophages.

### 2.5. The CaMKK2-, but Not the LKB1-, Dependent AMPK Signalling Pathway Contributes to the Activation of SIRT1-Mediated Autophagy and anti-Toxoplasma Effects in Primary Murine Macrophages

**Figure 5:**
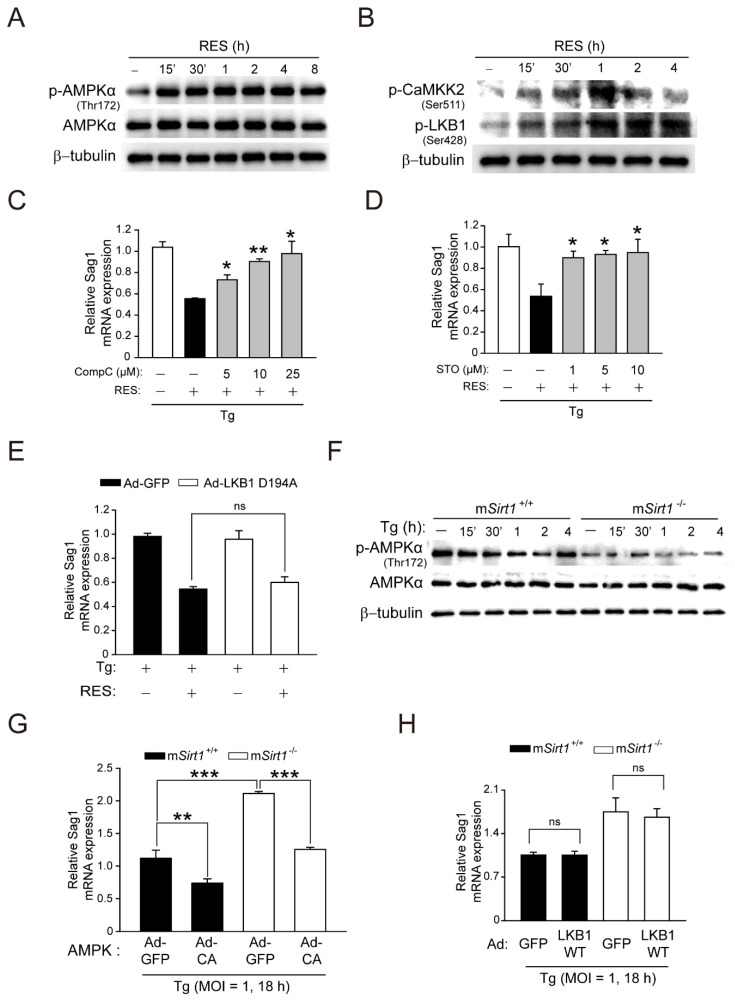
CaMKK2-dependent AMPK signalling is crucial for the activation of SIRT1-mediated antiparasitic responses against *Toxoplasma* infection in primary murine macrophages. (**A**,**B**) BMDMs were stimulated with RES (10 µM) for the various time periods, and then immunoblot analysis was assessed to evaluate the phosphorylation of AMPKα (for **A**) or CaMKK2 and LKB1 (for **B**). (**C**,**D**) BMDMs were pretreated with increasing concentrations of CompC (5, 10, or 25 μM, for C) or STO (1, 5, or 10 μM, for **D**) for 45 min, then infected with *T. gondii* RH strain (MOI = 1) for 18 h in the presence or absence of RES. *Sag1* mRNA expression was examined by qPCR analysis. (**E**) BMDMs transduced with Ad-GFP, Ad-LKB1 WT, or Ad-LKB1 D194A for 36 h (MOI of 10), respectively, were infected with a *T. gondii* RH strain (MOI = 1) for 2 h, then further incubated with RES (10 µM) for 22 h. *Sag1* mRNA expression was evaluated by qPCR analysis. (**F**) BMDMs from m*Sirt1^+/+^* and m*Sirt1*^−/−^ mice were infected with *T. gondii* for various time periods. Cell lysates were subjected to immunoblot analysis using antibodies against *p*-AMPKα (Thr172), AMPKα, and β-tubulin. (**G,H**) BMDMs from m*Sirt1^+/+^* and m*Sirt1*^−/−^ mice were transduced for 36 h with adenovirus-expressing GFP (Ad-GFP, for **G**,**H**), constitutively active AMPK (Ad-CA, for **G**), or WT LKB1 (Ad-LKB1 WT, for **H**), at a MOI of 10, and each cell then was infected with a *T. gondii* RH strain (MOI = 1) for 18 h. The mRNA expression of the *Sag1* gene was evaluated by real-time qPCR analysis. Data are representative of three independent experiments and are presented as means ± SD. * *p* < 0.05, ** *p* < 0.01, *** *p* < 0.001 (two-tailed Student’s *t*-test). Tg, *Toxoplasma gondii*; RES, resveratrol; CompC, Compound C; STO, STO-609; adenovirus-expressing GFP, Ad-GFP; adenovirus-expressing WT LKB1, Ad-LKB1 WT; adenovirus-expressing LKB1 D194A mutant, Ad-LKB1 D194A; ns, not significant.

CaMKK2-dependent AMPK signalling is essential for the docosahexaenoic acid (DHA)-induced activation of antimicrobial autophagy in *T. gondii* infection [13]. Importantly, SIRT1 and AMPK regulate each other’s activities, an imbalance in which is implicated in human diseases, such as cardiovascular disease, type 2 diabetes, inflammatory disease, and cancer [29]. We investigated whether AMPK is required for SIRT1-mediated autophagy activation and anti-*Toxoplasma* responses in primary macrophages. RES activated AMPKα within 15 min, an effect sustained for 8 h (Figure 5A). Moreover, the phosphorylation of CaMKK2 (Ser511) and LKB1 (Ser428), upstream kinases of AMPKα, was increased within 15 min of RES stimulation, which peaked at 1 h, in BMDMs (Figure 5B). Interestingly, the pretreatment of BMDMs with dorsomorphin compound C (CompC, a pharmacological AMPK inhibitor) or STO-609 (a selective inhibitor of CaMKK) significantly attenuated the RES-mediated increase in LC3 aggregates (Figure S2A). Moreover, the pretreatment of *T. gondii*-infected BMDMs with CompC (Figure 5C) or STO-609 (Figure 5D) significantly increased the mRNA level of *T. gondii Sag1*. However, the adenoviral transduction of a catalytically inactive form of LKB1 (Ad-LKB1 D194A; aspartic acid to alanine at residue 194) did not affect the RES-induced decrease in the *T. gondii Sag1* mRNA level (Figure 5E) or the RES-induced LC3-II protein level (Figure S2B).

We next examined whether a myeloid SIRT1 deficiency affects AMPK activation in response to *T. gondii* infection. *Sirt1*^−/−^ BMDMs exhibited the reduced phosphorylation of AMPKα under normal conditions and from 15 min to 4 h after *T. gondii* infection compared to m*Sirt1^+/+^* BMDMs (Figure 5F). The overexpression of adenovirus containing a constitutively active AMPK reduced the mRNA level of *T. gondii Sag1* in m*Sirt1^+/+^* and m*Sirt1*^−/−^ BMDMs (Figure 5G). However, adenoviral transduction with WT LKB1 in m*Sirt1^+/+^* and m*Sirt1*^−/−^ BMDMs did not alter the mRNA level of *T. gondii Sag1*. Collectively, these results implicate CaMKK2-AMPK, but not LKB1, signalling in SIRT1-mediated autophagy activation and anti-*Toxoplasma* responses.

### 2.6. SIRT1 Positively Regulates the Activity of FoxO3a Via AMPK Signalling Pathways

**Figure 6:**
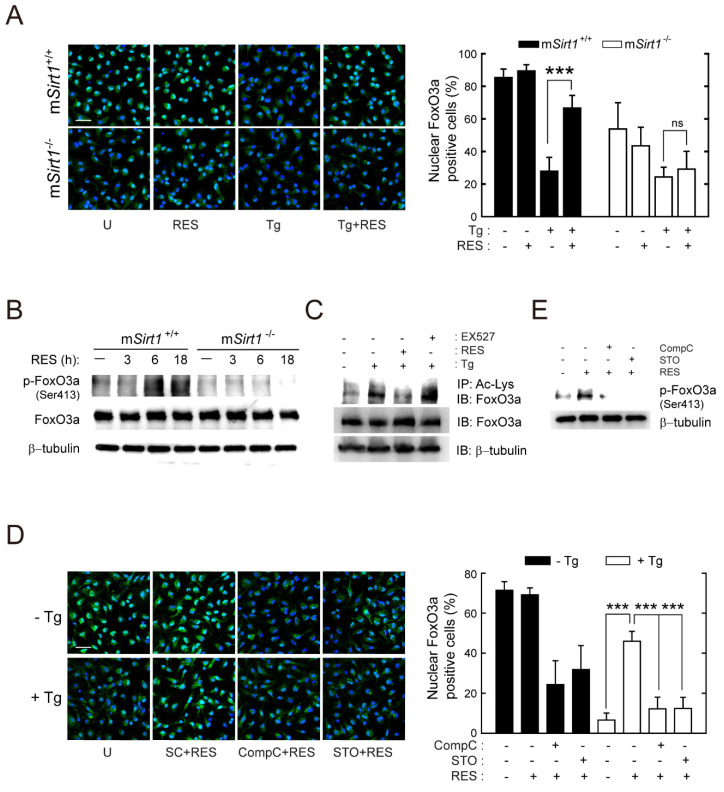
RES treatment promotes the activation of FoxO3a, which is mediated by SIRT1 and AMPK. (**A**) BMDMs from m*Sirt1^+/+^* and m*Sirt1*^−/−^ mice were infected with the RH strain (MOI = 1, for 2 h) and then further stimulated with RES (10 µM) for 18 h. Cells were fixed and immunostained with an anti-FoxO3 antibody (Alexa Fluor 488-conjugated goat anti-rabbit IgG, green) and DAPI for nuclei (blue). Cells were then subjected to immunofluorescence microscopy. Representative images (left) and quantitative data (right) showing the cytoplasmic translocation of FoxO3a were obtained from one representative of 3 independent samples, with each experiment containing a minimum of 50 cells scored in 7 random fields. Scale bar = 25 µm. (**B**) BMDMs from m*Sirt1^+/+^* and m*Sirt1*^−/−^ mice were stimulated with RES (10 µM) for the indicated time periods. The phosphorylation of FoxO3 (Ser413) was determined by immunoblot analysis. (**C**) BMDMs were infected with *T. gondii* for 18 h in the presence of RES (10 µM) or EX527 (4 µM). Cell lysates were subjected to immunoprecipitation analysis using an anti-acetylated-lysine antibody (Ac-Lys), and then the immunoprecipitates were analysed by Western blot using an anti-FoxO3a antibody. Cell lysates were harvested and subjected to Western blot analysis for FoxO3a and β-tubulin, as loading controls. (**D**) BMDMs were pretreated with Compound C (25 µM) or STO-609 (10 µM) for 45 min, then infected with RH strain (MOI = 1) for 18 h in the presence or absence of RES (10 µM). Cells were fixed, immunostained, and quantified as described in Figure 6A. Representative images (left) and quantitative data (right) showing the cytoplasmic translocation of FoxO3a. Scale bar = 25 µm. (**E**) BMDMs were pretreated with Compound C (25 µM) or STO-609 (10 µM, 45 min) and then stimulated with RES (10 µM, 18 h). The phosphorylation of FoxO3 (Ser413) was determined by immunoblot analysis. Data are representative of three independent experiments and are presented as means ± SD. *** *p* < 0.001 (two-tailed Student’s *t*-test). Tg, *Toxoplasma gondii*; RES, resveratrol; CompC, Compound C; STO, STO-609; ns, not significant.

The AMPK-mediated phosphorylation of FoxO3a transactivation domain sites results in the induction of FoxO3a nuclear accumulation and, subsequently, the transcription of target genes associated with autophagy activation [27,30]. To examine the role of SIRT1 in the regulation of FoxO3a activity during *T. gondii* infection, we enumerated cells with nuclear FoxO3a in RES-stimulated m*Sirt1^+/+^* and m*Sirt1*^−/−^ BMDMs. *Toxoplasma gondii* infection induced the cytosolic translocation of FoxO3a in m*Sirt1^+/+^* BMDMs, which was suppressed by RES (Figure 6A). Although FoxO3a cytoplasmic translocation after *T. gondii* infection in m*Sirt1*^−/−^ BMDMs was similar to that in normal cells, no inhibitory effect of RES was observed (Figure 6A). Indeed, RES induced the phosphorylation of FoxO3a (Ser413) within 6 h in m*Sirt1^+/+^* BMDMs, but not in m*Sirt1*-deficient BMDMs (Figure 6B). Moreover, *T. gondii*-induced FoxO3a acetylation was abolished by post-treatment with RES but was enhanced by the selective SIRT1 inhibitor EX-527 (Figure 6C).

Next, we investigated the involvement of AMPK signalling in SIRT1-mediated FoxO3a activation. Confocal images showed that RES-induced FoxO3a nuclear accumulation in *T. gondii*-infected cells was abolished by CompC or STO-609 (Figure 6D). Furthermore, CompC or STO-609 pretreatment significantly decreased FoxO3a nuclear accumulation (Figure 6D) and FoxO3a phosphorylation (Figure 6E) in BMDMs treated with RES (without infection by *T. gondii*). These findings indicate that CaMKK2/AMPK activation is essential for SIRT1-mediated FoxO3a activation during *T. gondii* infection.

## 3. Discussion

SIRT proteins are important players in human health and disease by regulating the cellular stress response [17,25]. *Neospora caninum* infection of caprine endometrial epithelial cells leads to mitochondrial dysfunction by downregulating SIRT1 expression, promoting the proliferation of intracellular protozoa [31]. In addition, SIRT3 activates the antimicrobial response to promote host survival by coordinating mitochondrial function and autophagy activation in *Mycobacterium tuberculosis* infection [32]. However, the mechanisms by which SIRT1 regulates the immune response to *T. gondii* are unclear.

Obligate intracellular parasites have several strategies to survive and proliferate in host immune and non-immune cells. During *T. gondii* infection, host-cell signalling cascades that inhibit autophagy targeting of PV can be activated, thereby preventing the constitutive autophagy-mediated lysosomal degradation of PV [14]. Therefore, drug candidates or endogenous factors capable of inducing autophagy have therapeutic potential for *Toxoplasma* infection. The correlation between SIRT1 activity and autophagy induction has been described [17]. We found that the pharmacological activation of SIRT1 by 4-HBA resulted in autophagy induction, which restricts the growth of intracellular parasites in primary murine macrophages [24]. In this study, a myeloid *Sirt1* deficiency increased susceptibility to *Toxoplasma* infection in mice and primary murine macrophages (Figure 1). Moreover, SIRT1 activation by RES suppressed intracellular parasite growth (Figure 2), the formation of autophagy vacuoles, the lipidation of the autophagy protein LC3, and autophagosome–lysosome fusion (Figure 3). However, the effect of RES on *T. gondii* infection was attenuated in m*Sirt1*^−/−^ BMDMs. Therefore, endogenous SIRT1 is important for the activation of a host immune response by regulating autophagy activity during *Toxoplasma* infection. Additional studies on the role of SIRT1 in various infection routes and strains of *T. gondii* and in immune cells important in *Toxoplasma* infection (e.g., neutrophils and dendritic cells) are needed.

SIRT1 modulates autophagy activity by deacetylating the transcription factors that regulate the expression of genes associated with the autophagy machinery, as well as by directly deacetylating Atg5, 7, and 8 in the cytoplasm [17,27]. FoxO transcription factors regulate autophagy at multiple levels, including transcription, by binding to the promoters of autophagy genes [33], directly interacting with autophagy proteins in the cytoplasm [34], and epigenetic modulation [35]. The transactivation activity of FoxO proteins is modulated by post-translational modification. Indeed, the AKT-dependent phosphorylation and acetylation of FoxO proteins promote their nuclear exclusion, whereas their AMPK-dependent phosphorylation and deacetylation lead to nuclear accumulation [27]. Herein, *T. gondii* infection of m*Sirt1^+/+^* BMDMs slightly increased the nuclear exclusion, phosphorylation, and acetylation of FoxO1, as well as the phosphorylation of AKT, compared to m*Sirt1*^−/−^ BMDMs. Moreover, *T. gondii*-induced nuclear exclusion of FoxO1 was attenuated by RES and PI3K/AKT inhibitor pretreatment in primary macrophages (Figure 4). Nuclear and cytoplasmic FoxO1 can induce autophagy, for which SIRT1 is essential. However, the mechanism of autophagy activation and cell fate (survival or autophagic death) differs depending on FoxO1 cellular localisation [17]. Specifically, the RES-induced pharmacological activation of SIRT1 in diabetic mice promoted the deacetylation of FoxO1, resulting in autophagy activation by binding to the Rab7 promoter [36]. By contrast, stresses such as starvation and oxidation strongly induce FoxO1 acetylation by p300 histone acetyltransferase and its dissociation from SIRT1. Furthermore, FoxO1 binding to Atg7 in the cytoplasm results in autophagic cell death [34,37]. Therefore, our results suggest that SIRT1 suppresses aberrant post-translational phosphorylation and acetylation and the nuclear exclusion of FoxO1 via PI3K-dependent AKT signalling during *T. gondii* infection, ultimately inducing autophagy.

Previous studies demonstrated that AMPK is an essential intermediator in the regulation of cellular energy homeostasis and the activation of host defenses against intracellular pathogens [38,39]. In *Toxoplasma* infection, CaMKK2-dependent AMPK signalling is required for CD40-mediated autophagic clearance [40]. Omega-3 polyunsaturated fatty acids promote the CaMKK2/AMPK-mediated induction of autophagy, which is crucial for protection against *T. gondii* infection [13]. In this study, RES rapidly activated CaMKK2-dependent AMPK signalling, which is required for autophagy activation and the suppression of intracellular parasitic growth. Moreover, *T. gondii*-induced AMPK phosphorylation was decreased in m*Sirt1*^−/−^ compared to m*Sirt1^+/+^* BMDMs. These data indicate that CaMKK2-dependent AMPK signalling is essential to the SIRT1-mediated activation of antiparasitic autophagy (Figure 5 and Figure S2). Because AMPK signalling is associated with the SIRT1-FoxO3 axis in autophagy activation and other physiological processes, we investigated the roles of these mediators in *Toxoplasma* infection. *Toxoplasma gondii* infection promoted the nuclear exclusion of FoxO3a in primary macrophages, but this effect was significantly inhibited by RES. Additionally, RES induced FoxO3a phosphorylation within 6 h in m*Sirt1^+/+^* BMDMs, but the effect was negligible in m*Sirt1*^−/−^ BMDMs. Importantly, the RES-induced nuclear accumulation of FoxO3a was suppressed by CompC or STO-609 pretreatment in primary macrophages, irrespective of *T. gondii* infection. AMPK activates FoxO3 phosphorylation at Ser413, promoting its stabilisation and nuclear accumulation and leading to the transcriptional or epigenetic activation of autophagy genes [35,41]. Our results suggest a correlation between AMPK and FoxO3a in the activation of the SIRT1-mediated antiparasitic response (Figure 6).

**Figure 7:**
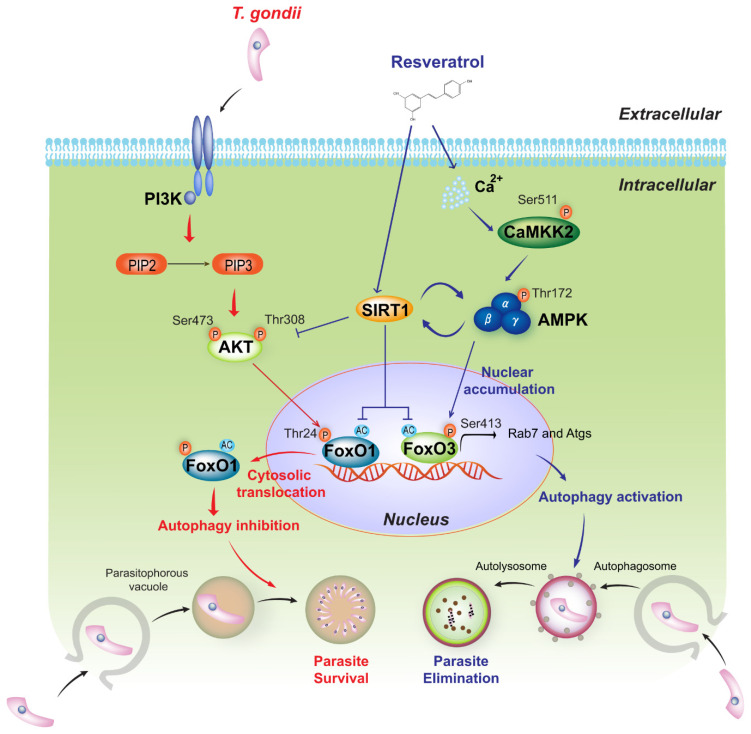
Essential function and molecular mechanism of SIRT1 in modulating the host immune response to *T. gondii* infection. In macrophages, myeloid-specific SIRT1 increases autophagy activity during *T. gondii* infection, thereby eliminating intracellular *T. gondii* by inducing the colocalisation of parasitophorous vacuoles with autophagosomes/lysosomes. As a key regulatory mechanism of autophagy, SIRT1 attenuates the acetylation of the transcription factor FoxO1 and its PI3K/AKT-dependent phosphorylation (Thr24) in response to *T. gondii*, thus regulating FoxO1 transactivation activity by preventing its nuclear exclusion. Moreover, SIRT1 promotes FoxO3a deacetylation and its CaMKK2/AMPK-dependent phosphorylation (Ser413), leading to nuclear accumulation and the transactivation of FoxO3a. Pharmacological activation of SIRT1 by RES induces antiprotozoal autophagy by regulating FoxO1 and FoxO3a activity via the PI3K/AKT and CaMKK2/AMPK signalling pathways, respectively.

In conclusion, myeloid-specific SIRT1 activates autophagy and the host response to *T. gondii* infection by controlling FoxO1 and FoxO3a transactivation activity via PI3K/AKT and CaMKK2/AMPK signalling, respectively. Moreover, the RES-dependent pharmacological activation of SIRT1 promotes antiparasitic autophagy, restricting the proliferation of *T. gondii* in primary murine macrophages (Figure 7). Our findings implicate SIRT1, in conjunction with the PI3K/AKT-FoxO1 and CaMKK2/AMPK-FoxO3a axis, in the activation of antiparasitic autophagy and will facilitate the development of novel therapeutics for *Toxoplasma* infection.

## 4. Materials and Methods

### 4.1. Mice and Ethics Statement

C57BL/6 and BALB/c mice were purchased from Koatech (Gyeonggi-do, Korea), and m*Sirt1*^−/−^ mice were kindly provided by Prof. Byung-Hyun Park (Department of Biochemistry, Chonbuk National University Medical School, Korea). Animal-related experimental procedures were approved by the Institutional Animal Care and Use Committee, Chungnam National University (202012A-CNU-200; Daejeon, Korea), and followed National Institutes of Health guidelines. Animals were housed in sawdust-covered cages in an air-conditioned environment under a 12 h light/dark cycle (5 animals per cage) with free access to standard rodent food and water. Animal breeding was provided by the staffs of IACUC and the Animal Core Facility under the guidance of supervisors who were certified animal technologists. Veterinary care was provided by the IACUC faculty and veterinary residents at the Chungnam National University School of Medicine.

### 4.2. Preparation of Cell and Parasite

BMDMs were differentiated over 5–7 days in a medium with a recombinant macrophage colony-stimulating factor (M-CSF), as described previously [42]. The culture medium consisted of Dulbecco’s modified Eagle’s medium (DMEM; Welgene, Gyeongsan, Korea) supplemented with 10% fetal bovine serum (FBS, Gibco BRL, Waltham, MA, USA) and 1% antibiotic–antimycotic (Gibco™ antibiotic–antimycotic (100X); Gibco BRL, Waltham, MA, USA). Human retinal pigment epithelial ARPE-19 cells (American Type Culture Collection, Manassas, VA, USA) were cultured in DMEM/F-12 (Welgene, Gyeongsan, Korea) with 10% FBS and 1% antibiotic–antimycotic.

The *T. gondii* RH strain was grown in ARPE-19 cells (MOI = 5) for 2–3 days at 37 °C and 5% CO_2_. Host cells and parasites were washed with phosphate-buffered saline (PBS) after spontaneous host cell disruption. Protozoans were suspended in cold medium and passed through a 26-gauge needle and a 5.0 μm pore filter (Millipore, Billerica, MA, USA). The GFP-RH strain was kindly provided by Dr. Yoshifumi Nishikawa (Obihiro University of Agriculture and Veterinary Medicine, Japan). The *T. gondii* ME49 strain was obtained from the brain tissue of BALB/c mice that infected 50 cysts and were maintained every 3 weeks.

### 4.3. Reagents and Antibodies

Resveratrol (RES; R5010), 3-methyladenine (3-MA; M9281), EX-527 (E7034), wortmannin (WM; W1628), and STO-609 were purchased from Sigma-Aldrich (Saint Louis, MO, USA), and Compound C (171260) was purchased from Calbiochem (San Diego, CA, USA). SYBR Green reagents were purchased from Applied Biosystems (Waltham, MA, USA). Dimethyl sulfoxide (DMSO; Sigma-Aldrich, Saint Louis, MO, USA) was used at 0.1% (*v/v*) as a solvent control.

Specific antibodies against AMPKα (2532), FoxO3a (75D8), FoxO1 (C29H4), phospho-Fox01 (9464), phospho-AMPK (2535), phospho-CAMKK2 (12818), phospho-LKB1 (3482), and acetylated-Lysine (9441) were purchased from Cell Signaling (Danvers, MA, USA). Phospho-Fox03a (AF2343) was purchased from Affinity Biosciences. Anti-LC3 (L8918) was obtained from Sigma-Aldrich. Anti-β-tubulin (Ab6046) and anti-SIRT1 (Ab28170) were purchased from Abcam (Burlingame, CA, USA). Anti-TP3 (sc-52255) and anti-LAMP1 (sc-17768) were purchased from Santa Cruz (Dallas, TX, USA). All other reagents were obtained from Sigma-Aldrich, unless otherwise indicated.

### 4.4. Immunoprecipitation and Western Blot Analysis

For immunopreciptiation, cells were washed twice with ice-cold PBS and lysed in NP-40 buffer (20 mM Tris (pH 7.5), 135 mM NaCl, 2 mM EDTA, 10% glycerol, 1% NP-40) with protease inhibitors and phosphatase inhibitor cocktail (Roche) for 30 min. Each lysate were centrifugated at 15,000× *g* at 4 °C for 15 min and proteins (500 mg) were immunoprecipitated using specific antibodies. Protein G sepharose beads (GE Healthcare, Piscataway, NJ, USA) were then included in each sample and incubated for 2 h at 4 °C. Samples were subjected to Western blot analysis.

For Western blot analysis, cells were collected and lysed in PRO-PREP (iNtRON Biotechnology) with various protease inhibitors. Protein concentrations were evaluated with a BCA assay kit. Proteins (30 µg) were immediately heated at 100 °C for 5 min, separated by SDS-PAGE, transferred to polyvinylidene fluoride membranes (Millipore, Billerica, MA, USA). Membranes were developed using a chemiluminescence assay kit (Millipore) and analysed by Alliance Mini HD6 (UVitec, Cambridge, MA, USA).

### 4.5. RNA Extraction, Semi-Quantitative RT-PCR, and Real-Time Quantitative PCR

Total RNA was isolated using Trizol reagent (Invitrogen, Waltham, MA, USA) according to the manufacturer’s instructions, and semi-quantitative RT-PCR and real-time quantitative PCR were performed as described previously [42]. Briefly, cDNA was synthesised using Reverse Transcriptase Premix and amplified using Solg^TM^ 2X Taq PCR Pre-Mix (Solgent, Daejeon, Korea). The products were resolved on 1% agarose gel for semi-quantitative RT-PCR or were analysed using the StepOne™ and StepOnePlus™ Software (Applied Biosystems, Waltham, MA, USA) for real-time quantitative PCR. All primers used in this study were follows: *Sag1* (forward: 5′-ATCGCCTGAGAAGCATCACT-3′; reverse: 5′- GCGAAAATGGAAACGTGACT-3′), *β-actin* (forward: 5′- TCATGAAGTGTGACGTTGACATCCGT-3′; reverse: 5′-CCTAGAAGCATTTGCGGTGCACGATG-3′).

### 4.6. Experimental Murine Toxoplasmosis Model

To establish an in vivo toxoplasmosis model, mice were intraperitoneally (i.p.) infected with 40 cysts of strain ME49. Cysts in the mice brain homogenate were obtained at 3 weeks. The number of tissue cysts was counted under a light microscopy. To analyse the mRNA expression of the *Sag1* gene, cDNA synthesis was carried out using Reverse Transcriptase Premix. cDNA was subjected to amplification using a real-time PCR instrument.

### 4.7. Adenovirus Production

Adenoviral vectors were used for target gene overexpression or mutation. Ad-GFP, Ad-DN-AMPK, and Ad-CA-AMPK were kindly provided by Dr. Hueng-Sik Choi (Chonnam National University, Gwangju, Korea). Ad-LKB1 and Ad-LKB1 D194A were kindly provided by Dr. Don-kyu Kim (Chonnam National University) [43]. Large-scale adenovirus production was achieved as described previously [42,44]. Briefly, HEK293A cells were transfected with adenovirus (MOI = 10), and the adenovirus-containing cells and media were subjected to three freezing (–196 °C) and thawing (37 °C) cycles. After centrifugation at 3000 rpm for 15 min, Ad-DN-AMPK and Ad-CA-AMPK were concentrated by CsCl gradient ultracentrifugation, and Ad-DN-LKB1 and Ad-LKB1 were purified using Adeno-X Maxi Purification Kit (Clontech) per the manufacturer’s protocol. Then, the viral titers were evaluated using an Adeno-X Rapid Titer Kit (Clontech, Mountain View, CA, USA).

### 4.8. Quantification of Intracellular T. gondii

BMDMs were seeded on 22-mm glass coverslips and then infected with the GFP-RH strain for the indicated time periods. The coverslips were washed using warmed PBS, fixed with 4% paraformaldehyde in PBS for 10 min, and permeabilised with 0.25% Triton X-100 in PBS for 10 min. Texas Red^®^-X phalloidin (Life Technologies Corporation, Carlsbad, CA, USA) for cytosol and 4′6-diamidino-2-phenylindole (DAPI, Sigma) for nucleus were used. Cover slides were analysed by confocal laser scanning microscopy (Leica TCS SP8, Leica microsystems).

### 4.9. Immunofluorescence Analyses

Immunofluorescence analysis of endogenous LC3 puncta and nuclear translocation analysis of FoxO1 and FoxO3 was performed as described previously [45]. Briefly, after fixation and permeabilisation, cells in coverslips were stained with a specific antibody for LC3 (1:400; MBL International, PM036), FoxO3a, or FoxO1 for 2 h at room temperature. After washing excess primary antibody with PBS, cells were incubated with Alexa Fluor 488-conjugated goat anti-rabbit IgG (molecular probes) or Alexa Fluor 568-conjugated goat anti-rabbit IgG (molecular probes) for 2 h.

### 4.10. Analysis of Autophagosome Maturation and Lysosomal Fusion with PV

For analysis, the autophagosome maturation and lysosomal fusion with PV, BMDMs were cultured on 22 mm glass coverslips and infected with the GFP-RH strain. The cells were fixed with 4% paraformaldehyde in PBS for 10 min and permeabilised with 0.25% Triton X-100 in PBS for 15 min. Cells in coverslips then were stained with specific antibody for LC3 (1:400; MBL International, PM036) and LAMP1 (1:400) overnight at 4 °C. After washing the samples with PBS, it were incubated with Alexa Fluor 488-conjugated goat anti-rabbit IgG or Alexa Fluor 594-conjugated goat anti-rabbit IgG for 2 h. After mounting, fluorescence images were acquired with a confocal laser-scanning microscope.

### 4.11. Statistical Analysis

A two-tailed Student’s *t* test was used to analyse differences between independent experimental data (means ± standard deviation [46] or the means ± standard error of the mean [47]). Differences were deemed significant at *p* value under 0.05.

## Data Availability

Not applicable.

## Supplementary Materials

The following supporting information can be downloaded at: https://www.mdpi.com/article/10.3390/ijms232113578/s1.
