# Supplementary material for: SIRT1 Promotes Host Protective Immunity against Toxoplasma gondii by Controlling the FoxO-Autophagy Axis via the AMPK and PI3K/AKT Signalling Pathways
Source: Int J Mol Sci. 2022 Nov 5;23(21):13578. doi: 10.3390/ijms232113578 (PMC9654124; doi:10.3390/ijms232113578)
Supplement: Supplementary file 1 [file ijms-23-13578-s001.zip › ijms-1905762-supplementary.pdf]

International Journal of *Molecular Sciences*

MDPI

Article

SIRT1 Promotes Host Protective Immunity against *Toxoplasma gondii* by Controlling the FoxO-Autophagy Axis Via the AMPK and PI3K/AKT Signalling Pathways

Jina Lee [1,2,3,†], Jinju Kim [1,2,3,†], Jae-Hyung Lee [1,2,3], Yong Min Choi [1,2,3], Hyeonil Choi [1], Hwan-Doo Cho [1], Guang-Ho Cha [1,2], Young-Ha Lee [1,2], Eun-Kyeong Jo [2,3,4], Byung-Hyun Park [5] and Jae-Min Yuk [1,2,3,*]

1 Department of Infection Biology, College of Medicine, Chungnam National University, Daejeon 35015, Korea; leejinah1988@gmail.com (J.L.); w1l2s3@naver.com (J.K.); dlwogud1003@naver.com (J.-H.L.); ymcdragon@naver.com (Y.M.C.); perfectviewman@naver.com (H.C.); johwandoo@naver.com (H.-D.C.); gcha@cnu.ac.kr (G.-H.C.); yhalee@cnu.ac.kr (Y.-H.L.)

2 Infection Control Convergence Research Center, College of Medicine, Chungnam National University, Daejeon 35015, Korea; hayoungj@cnu.ac.kr

3 Department of Medical Science, College of Medicine, Chungnam National University, Daejeon 35015, Korea

4 Department of Microbiology, College of Medicine, Chungnam National University, Daejeon 35015, Korea

5 Department of Biochemistry, Chonbuk National University Medical School, Jeonju 54896, Korea; bhpark@jbnu.ac.kr

* Correspondence: yjaemin0@cnu.ac.kr

† These authors contributed equally to this work.

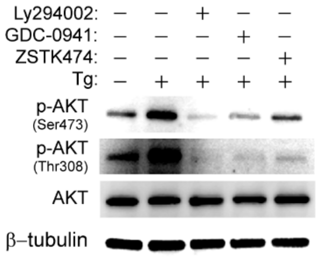

Figure S1. *T. gondii* infection induces the phosphorylation of AKT in primary macrophages and mice. BMDMs were infected with *T. gondii* for 18 h in a presence of LY294002 (10 μM), GDC0941 (250 nM), or ZSTK474 (10 nM). The phosphorylation of AKT at the residues of Ser473 and Thr308 was evaluated by immunoblot analysis and total protein was determined by monitoring β-tubulin and AKT, as a loading control. Tg, *Toxoplasma gondii*.

A

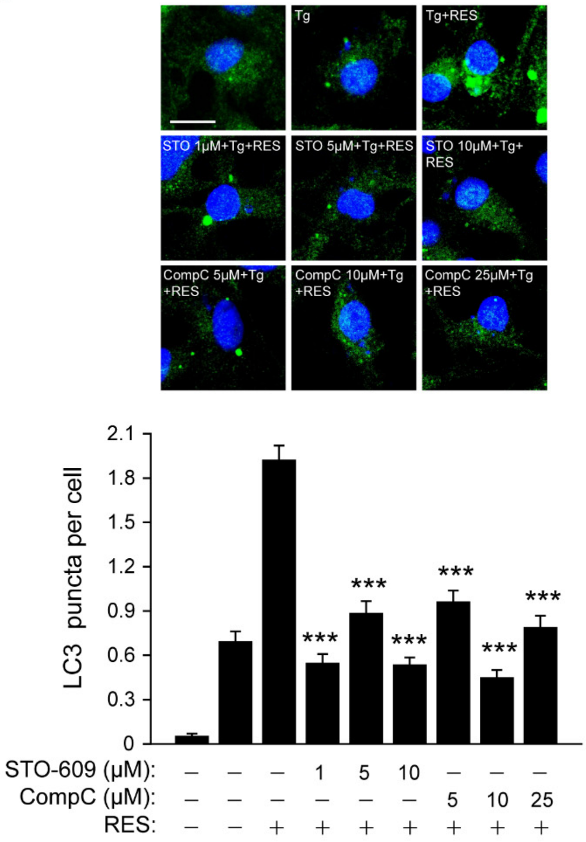

B

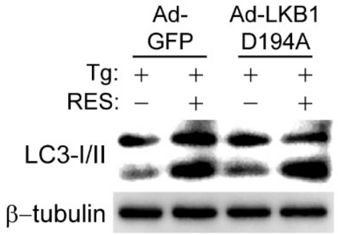

Figure S2. RES treatment activates autophagy induction through CaMKK2-dependent, not LKB1-dependent AMPK signaling in primary macrophages infected with *T. gondii*. (**A**) BMDMs were pretreated with increasing concentrations of the AMPK inhibitor Compound C (5, 10, or 25 μM) or the CaMKK2 inhibitor STO-609 (1, 5, or 10 μM) for 45 min, and then infected with *T. gondii* for 18 h in a presence or absence of RES (10 μM). Cells were subjected to immunofluorescence microscopic analysis of LC3 puncta formation (top). Quantitative analysis of LC3 punctate foci per cell (bottom). Each experiment included at least 100 cells scored in 5 random fields. Scale bar = 10 μm. (**B**) BMDMs transduced for 36 h with adenovirus expressing GFP (Ad-GFP) or LKB1 D194A mutant (Ad-LKB1 D194A), at a MOI of 10, were infected with *T. gondii* RH strain (MOI = 1) for 2 h, and then incubated with RES (10 μM) for 22 h. Total cell lysates were subjected to immunoblot analysis to determine the protein expression of LC3 and β-tubulin. Data are representative of three independent experiments and are presented as means ± SD. *** $p < 0.001$ (two-tailed Student's *t*-test). Tg, *Toxoplasma gondii*; RES, Resveratrol; CompC, Compound C; STO, STO-609.
